# Graphene Oxide-Polypyrrole Coating for Functional Ceramics

**DOI:** 10.3390/nano10061188

**Published:** 2020-06-18

**Authors:** Nelly Ma. Rosas-Laverde, Alina Iuliana Pruna, David Busquets-Mataix

**Affiliations:** 1 Department of Materials, National Polytechnic University, Quito 170517, Ecuador; Nelly.rosas@epn.edu.ec; 2 Center for Surface Science and Nanotechnology, Polytechnic University of Bucharest, 060042 Bucharest, Romania; 3 Institute of Materials Technology, Universitat Politècnica de València, 46022 Valencia, Spain; dbusquets@mcm.upv.es

**Keywords:** ceramic, NiMoP electroless deposition, electrodeposition, graphene oxide, polypyrrole, supercapacitor

## Abstract

Ceramic substrates were metallized with a Ni-Mo-P electroless coating and further modified with a polypyrrole (PPy) coating by the electrodeposition method. The properties of the polypyrrole coating were studied with the addition of a graphene oxide (GO) nanomaterial prior to the electrodeposition and its reduction degree. Fourier Transform Infrared Spectroscopy, Field-Emission Scanning Electron Microscopy, Raman spectroscopy and cyclic voltammetry were employed to characterize the properties of the coatings. The results indicated the successful synthesis of conductive electrodes by the proposed approach. The electrodeposition of PPy and its charge storage properties are improved by chemically reduced GO. The surface capacitive contribution to the total stored charge was found to be dominant and increased 2–3 fold with the reduction of GO. The chemically reduced GO-modified PPy exhibits the highest capacitance of 660 F g^−1^ at 2 mV s^−1^, and shows a good cyclability of 94% after 500 charge/discharge cycles. The enclosed results indicate the use of an NiMoP electroless coating, and modification with a carbon nanomaterial and conducting polymer is a viable approach for achieving functional ceramics.

## 1. Introduction

From the point of view of low-cost production, environmentally friendliness, and performance, renewable energy production and energy storage are among the most popular and interesting worldwide areas. In this respect, the design of ceramics for next-generation energy storage devices emerged as a field ripe with venues for new materials. Ceramic substrates have attracted much research interest thanks to their chemical and environmental stability, low cost, and good mechanical and thermal properties. In order to overcome the major drawback of their insulating nature [1], the electroless deposition (ED) was proposed as a promising approach as it is a facile, low cost, and efficient process allowing the synthesis of conductive, homogenous, continuous, and compact films irrespective of the geometry and nature of the substrate, without the need for external electricity [2,3,4,5,6,7,8,9].

Currently, supercapacitors have attracted significant interest as next-generation energy storage devices bridging batteries and capacitors [10,11], thanks to their properties such as high power density, longer cycling performance, and low environmental pollution [12,13,14]. The total charge stored in the electrode is generally based on the surface and bulk processes, which involve, on one hand, two different mechanisms such as surface redox and ion adsorption/desorption reactions or electric double layer formation at the electrode/electrolyte interface and, on the other, a diffusion-controlled intercalation process [13,15,16]. Thus, a combination of these mechanisms is often pursued to obtain hybrid supercapacitors with improved performance. However, given the complex nature of the electrode materials developed lately, the definition of their storage mechanisms is of paramount importance. In this respect, there are many reports on the deconvolution of surface and bulk electrochemical processes in energy storage electrodes [17,18,19].

The materials with a high surface area and electrical conductivity are highly employed in energy storage devices, as it is known that electrical double-layer capacitors (EDLCs)-based electrodes mainly involve charge stored via electrostatic force at the electrode/electrolyte interface [13]. These materials include carbon nanomaterials, such as carbon nanotubes, graphene and its oxide derivatives, carbon fibers, etc. [20,21,22]. Currently, reduced graphene oxide (rGO) is the most used carbon nanomaterial in supercapacitor electrodes due to its excellent thermal, electrical, and mechanical properties [23]; cycling stability; and large potential window [24]. The rGO can be obtained from GO by thermal, photothermal, chemical, or electrochemical reduction methods [25]. On the other hand, oxide and hydroxide materials such as MnO_2_, NiO, Co(OH)_2_ or conducting polymers are usually employed for pseudo-capacitance, as they exhibit fast and reversible Faradaic processes between electrode materials and electrolytes [13,15]. Polypyrrole (PPy) is seen as the most promising alternative conductive polymer for supercapacitor electrode material due to its advantages, such as its simple and easy synthesis, low cost, excellent capacitance, and environmental compatibility [26].

This work proposes a two-step approach based on the combination of electroless deposition and electrodeposition methods for the modification of ceramic electrodes towards functional ceramics. A conductive NiMoP coating deposited onto the ceramic surface serves as an electrode for the deposition of PPy by the cyclic voltammetry (CV) method. GO is employed for the further surface modification of the ceramic, and the effect of its reduction on the electrodeposition of PPy is assessed. The properties of the obtained electrodes for energy storage are evaluated by CV, as this method provides with a simple yet effective technique by which to electrochemically evaluate the reaction kinetics of active materials in order to deconvolute the contribution of surface and bulk electrochemical processes to the total stored charge. The obtained results indicate the successful fabrication of a simple, fast, and low-cost ceramic supercapacitor electrode which exhibits a good performance (about 660 F g^−1^ at 2 mV s^−1^) and capacitance retention of 94% after 500 charge/discharge cycles.

## 2. Materials and Methods

### 2.1. Materials

All the chemicals (Alfa Aesar, Kandel, Germany) were reagent grade and used as received. A graphene oxide aqueous dispersion (Sigma Aldrich, Madrid, Spain) and pyrrole monomer (Sigma Aldrich, Madrid, Spain), which was distilled prior to usewere employed for the coatings. The ceramic substrates (5 cm × 1.5 cm × 0.3 cm) were fabricated in the laboratory using porcelain stoneware atomized powder obtained by mixing 59.85% SiO_2_, 15.99% Al_2_O_3_, 0.48% Fe_2_O_3_, 4.87% CaO, 3.59% of MgO, 1.45% Na_2_O, 1.30% K_2_O, and 0.61% Ti_2_O (wt. %) [1].

### 2.2. Fabrication of PPy/rGO Ceramic-Supported Electrodes

#### 2.2.1. Metallization of Ceramic Substrate by Electroless Process

First, the ceramic substrates were metallized by the electroless deposition (ED) process according to the method developed by Rosas-Laverde et al. [1]. Briefly, the ceramic substrate was airbrushed with the Pd catalyst solution and subjected to thermal activation at 300 °C for 12 h. Then, the ceramic substrate was immersed in the NiMoP plating bath at 80 °C for 30 min. Finally, the substrate was washed with distilled water and air-dried. The obtained substrates were denominated as metallized ceramic substrates (CS). A detailed characterization of the electroless deposition process and obtained coating is available elsewhere [1].

#### 2.2.2. Modification of CS Substrates with GO

Prior to use, the GO dispersion (0.5 mg mL^−1^) was sonicated for 1 h. The CS substrates were modified with a GO layer by the dip-coating method, employing 3 sequences of 5 min immersion in an ultrasonic bath, washing with distilled water, and drying at 60 °C for 10 min. Finally, the GO-modified CS substrates were dried at 60 °C for 2 h.

#### 2.2.3. Reduction of GO

CS substrates modified with reduced graphene oxide (rGO) were obtained by electrochemical and chemical methods. The electrochemical reduction was carried out by the CV method in 0.1 M of KCl solution using the GO-modified CS as a working electrode. The CV was performed in the −1.4 and 0 V potential range vs. Ag/AgCl at 50 mV s^−1^ for 10 cycles [15]. The obtained electrode was named rGO_E_.The chemical reduction of GO was performed using L-ascorbic acid in an ascorbic acid:GO w/w ratio of 10:1 at 60 °C for 10 h [27]. The obtained rGO substrates were rinsed with distilled water and air-dried. The obtained electrode was named rGO_C_.

#### 2.2.4. Electrodeposition of PPy on rGO-Modified CS Substrates

The PPy layer was electropolymerized onto CS substrates by the CV method from a solution of 0.1 M Py, 20 mM sodium dodecyl sulfate (SDS), and 0.05 M of sodium para toluene sulfonate (NapTS) at an ambient temperature [15]. The CS substrates without and with GO-based coatings were used as working electrodes. The CV deposition was performed between 0 and 1 V vs. Ag/AgCl at a scan rate of 50 mV s^−1^ for 30 cycles. Finally, the substrates were rinsed with distilled water and dried at 60 °C for 12 h. The obtained electrodes were named PPy, PPy/GO, PPy/rGO_E_, and PPy/rGO_C_-modified CS electrodes.

### 2.3. Characterization

The morphology of the coatings was characterized by Field Emission Scanning Electron Microscopy (FE-SEM) using a Bruker microscope working at a 2 kV and Atomic Force Microscopy (AFM) using a Bruker Multimode 8 microscope in tapping mode. X-ray diffraction (XRD) was performed using a Rigaku Ultima IV diffractometer in the Bragg–Bentano configuration using Cu Ka radiation (CuKα = 1.54 Å). Fourier transform infrared spectroscopy (FTIR) was performed on a Spectrum 100 (Perkin Elmer) spectroscope, while the Raman spectroscopy was performed on a LabRam HR UV spectroscope (Horiba) using a He-Ne (632.8 nm) laser with a 1.6 cm^−1^ resolution. The electrical resistivity of the NiMoP coatings on the ceramic substrate was determined by the Hall effect using a four-point probe (Ecopia, HMS-3000) at room temperature in a constant magnetic field of 1 T [28,29]. All the electrochemical experiments were carried out by using a potentiostat (PGSTAT 101, AUTOLAB) and NOVA software (version 1.11, Metrohm Autolab, Utrecht, The Netherlands) in a conventional three-electrode electrochemical cell using the modified CS substrates as working electrodes, Pt foil as a counter-electrode, and Ag/AgCl in saturated KCl as a reference electrode. The electrochemical performance for energy storage was assessed by CV measurements in 0.5 M Na_2_SO_4_ solution in the potential range from −0.1 to 0.7 V vs. Ag/AgCl at varying scan rates from 2 to 100 mV s^−1^. The CV stability was evaluated between −0.1 and 0.7 V vs. Ag/AgCl at a scan rate of 50 mV s^−1^ for 500 cycles. The areal capacitance *C_A_* (F cm^−2^) was calculated using the equation *C_A_ = ∫ i dV/(ν × ΔV × A)*, where *i* is the current, *ν* is the scan rate, *ΔV (V)* is the potential window, and *A* is the apparent area of the electrode [30,31].

## 3. Results and Discussion

**Figure 1:**
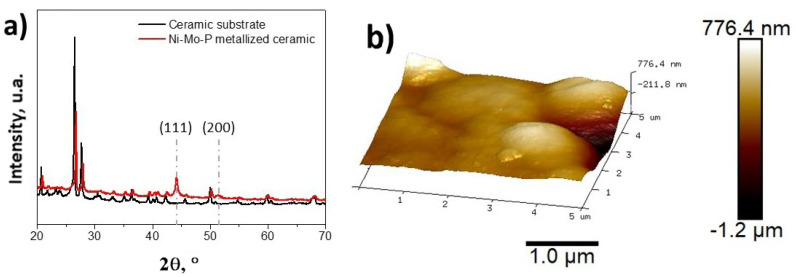
X-ray diffraction pattern (**a**) and Atomic Force Microscopy (AFM) images (**b**) of the NiMoP coating.

First, the metallization of the ceramic surface was analyzed by the XRD and AFM techniques. Figure 1 shows the crystalline phases and morphology of the NiMoP coating deposited onto the ceramic substrate by the electroless process.

The characteristic peaks of the NiMoP coating are clearly identified and they are located at about 44° and 51°, being attributed to the Ni (111) and Ni (200) reflection planes, respectively, in agreement with other reports [1]. The 3D AFM image of the NiMoP coating is shown in Figure 1b and indicates that the NiMoP film consists in fine spherical grains [1,32]. The AFM measurements indicated a *Ra* roughness of 59.0 ± 5.9 nm and a thickness of 465.3 ± 16.5 nm for the obtained NiMoP coating. The Hall measurements indicated a volume resistivity of 10.6 ± 2.2 μΩ cm for the metallized ceramic substrates.

**Figure 2:**
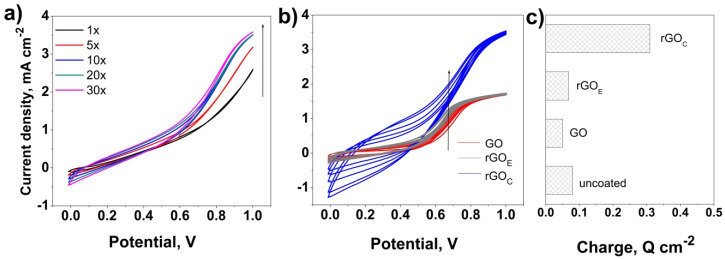
Cyclic voltammetry of polypyrrole (PPy) at the surface of ceramic substrates (CS) before (**a**) and after modification with graphene oxide (GO)-based films (**b**); evolution of the PPy deposition charge with the coating on the CS substrates (**c**).

The metallized ceramic substrates were further dip-coated in order to be modified with GO-based nanomaterials. The effect of the GO reduction degree was studied by chemical reduction with ascorbic acid and electrochemical reduction by cyclic voltammetry, as referenced elsewhere [15]. The evolution of CV curves for the PPy electrodeposition at the surface of the NiMoP-coated ceramic electrode is presented in Figure 2 as a function of the deposition cycles before and after the modification with GO and reduction of the GO films, namely rGO_E_ and rGO_C_. Figure 2a reveals the initiation of electropolymerization at a potential of about 0.7 V, which corresponds to the oxidation of the pyrrole monomers (Py) into Py radicals to form a PPy matrix in agreement with other reports, thus confirming the appropriate electric properties of the NiMoP coating to achieve PP electrodeposition [33]. The polymerization potential further shifts to lower values and the current response increases faster upon cycling, which corresponds to the growth of the PPy nanostructures—that is, the thickness of the coating increases (Figure 2b). Upon the modification of the ceramic substrate with GO, the polymerization current markedly decreases, as can be seen in Figure 2b, which is due to the insulating nature of GO. However, it is noted that the electrodeposition of the PPy coating is still allowed by the GO film being thin enough. The PPy electropolymerization potential slightly shifts to lower potential values upon the reduction of GO. The chemical reduction of GO appears to allow a higher shift of the polymerization potential up to 0.55 V, while the current response markedly increases, reaching a similar value with the process developed on the unmodified ceramic in Figure 2a. This evolution is attributed to the increase in the conductivity of rGO as more oxygen groups of GO are removed, while the residual ones can act as nucleation sites to electropolymerize the PPy matrix [34]. Figure 2c, depicting the average polymerization charge evolution as a function of the electrode, further confirms the marked effect of the GO nature on the electrodeposition of PPy, as the deposition charge markedly increases upon the reduction of GO due to the higher electrical conductivity of GO [35].

**Figure 3:**
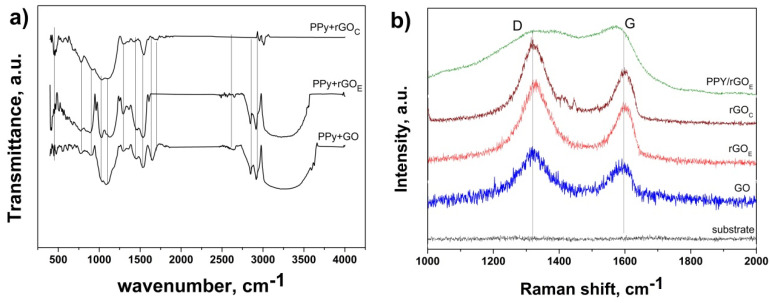
FTIR (**a**) and Raman (**b**) spectra of the coatings at the surface of the NiMoP coated ceramic.

The fabrication of the PPy-based coatings and the identification of different functional groups were assessed by FTIR and Raman spectroscopy, as depicted in Figure 3. The FTIR analysis (Figure 3a) revealed similar features for all the coatings, however a few differences were noted which are attributed to the reduction of GO. Thus, the GO-based coating shows a broad peak in the range of 3600 to 2900 cm^−1^ which is due to the carboxyl O–H stretching mode [36,37], two peaks located at 2930 and 2859 cm^−1^ which are assigned to the asymmetric and symmetric CH_2_ stretching of GO, a peak located at 1630 cm^−1^ assigned to C=C stretches from an unoxidized graphitic domain [38,39], a peak at around 1704 cm^−1^ which is attributed to the C=O stretching of the carboxyl group [40,41], a peak at 1440 cm^−1^ assigned to epoxy C–O [42], a peak at 1295 cm^−1^ corresponding to the C–OH stretching of the alcohol group [37], and one located at 1033 cm^−1^ which is attributed to the C–O stretching vibrations of the carboxyl groups [43,44]. While the coating based on the electrochemical reduction of GO resulted in similar FTIR features to the GO-based one, the one obtained from the chemical reduction shows no wide band at 2900–3600 cm^−1^ and less intense bands at 2930, 2859, and 1033 cm^−1^, which indicates a higher reduction degree. On the other hand, the presence of PPy in the coatings is indicated by the FTIR peaks located at 782 and 905 cm^−1^, the band emerging at 1295 cm^−1^ corresponds to the C−N characteristic stretching vibrations, the one at 1104 cm^−1^ is attributed to N–H in-plane deformation vibration, and the one at 1535 cm^−1^ is attributed to the C–N stretching in the pyrrole ring [34,35,45].

The Raman spectra of the PPy/rGO coatings at the surface of the metalized ceramic are depicted in Figure 3b. The GO layer exhibits the typical Raman bands located at 1319 and 1519 cm^−1^, representing the D and G bands which are associated with structural imperfections and disorder in the graphitic structure and the sp2 hybridized carbons, respectively [46]. Upon reduction by either the chemical or electrochemical method, the intensity ratio I_D_/I_G_ was affected—namely, the I_D_/I_G_ increased from 1.09 corresponding to the GO coating up to 1.33 corresponding to the GO coating reduced by the chemical approach. The position of the D bands shifted from 1318 for the GO layer to 1324 cm^−1^ corresponding to the GO coating reduced by the chemical approach, which suggests an improved reduction degree by the chemical reduction approach with respect to the electrochemical one. On the other hand, the growth of PPy at the surface of the modified GO ceramics is confirmed from the appearance of new bands located at 1370 cm^−1^, which is attributed to the ring stretching, and 1400 cm^−1^ [47,48], along with the evolution of the typical GO bands, where the shift in the position of the G peak to 1580 cm^−1^ corresponding to the PPy/rGO_C_ composite coating indicates a marked coupling and charge transfer between the PPy and rGO [49].

**Figure 4:**
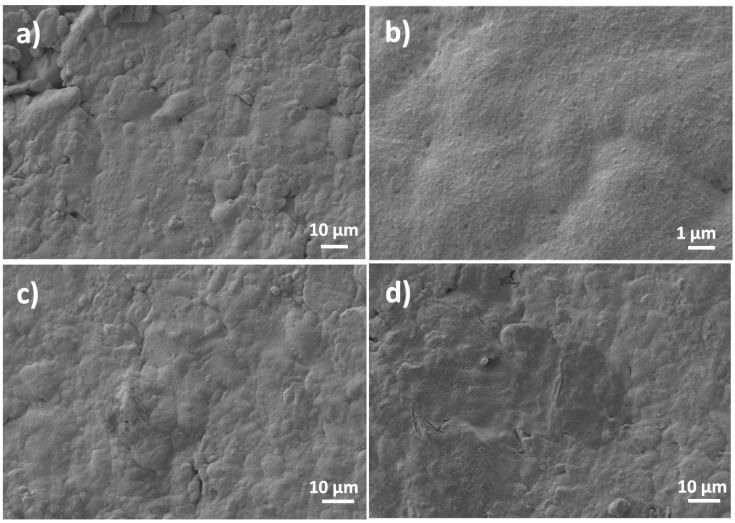
FE-SEM images of the NiMoP electroless coating before (**a**,**b**) and after modification with GO reduced by the electrochemical approach (**c**) and the chemical approach (**d**).

Figure 4 shows the FE-SEM images of the NiMoP coated ceramic before and after the modification with GO film reduced by the chemical and electrochemical methods. As can be seen in Figure 4a,b, the NiMoP coating exhibits a good continuity and homogeneity. The presence of rGO films at the surface of NiMoP is indicated in Figure 4c,d by the smoother aspect of the electroless coating and some darker areas, which are attributed to agglomerated rGO sheets.

**Figure 5:**
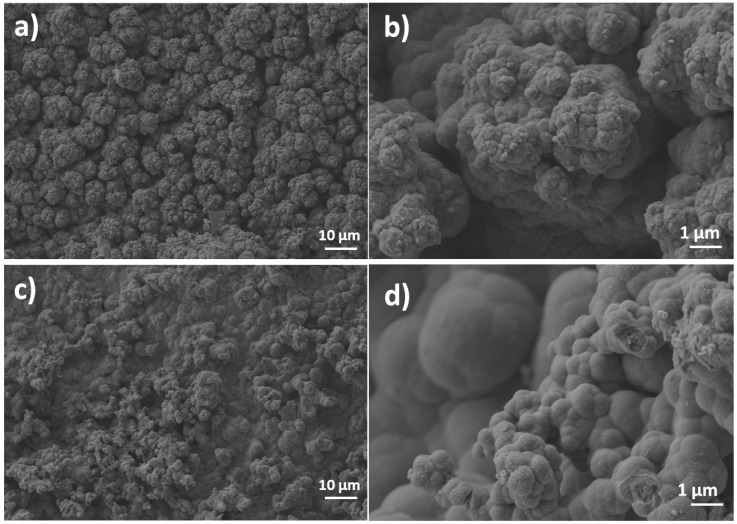
FE-SEM images of the deposition of PPy onto CS electrodes modified with electroreduced GO (rGO_E)_ (**a**,**b**) and chemically-reduced GO (rGO_C_) (**c**,**d**).

The electrodeposition of PPy onto the rGO_E_–CS and rGO_C_–CS electrodes is depicted in Figure 5. The FE-SEM images indicate successful electropolimerization by the formation of a continuous PPy film exhibiting the typical globular morphology of this kind of polymer in both cases of substrates [33]. The thickness of the coatings was determined from section SEM images and reached 500 nm ± 30 nm for the one deposited on rGOE, while the one deposited on rGOC exhibited a thickness of 620 ± 50 nm. The micrographs obtained at a higher magnification reveal a better definition and order of the PPy globules in the case of the rGO_C_–CS electrode than for the rGO_E_–CS one, indicating a dependence of the electrodeposited PPy morphology on the GO reduction in agreement with the electrochemical results in Figure 2.

**Figure 6:**
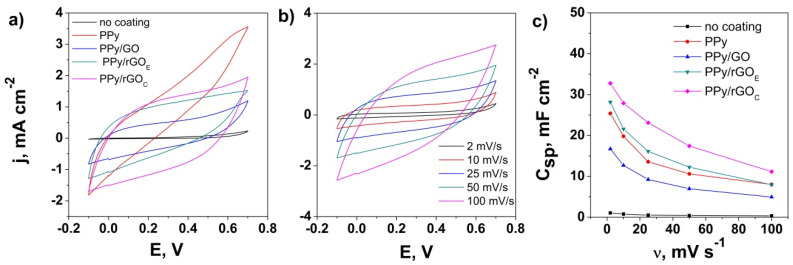
CV evolution of the PPy with coating on the CS substrate (**a**), cyclic voltammetry (CV) curves of the PPy/rGO_C_–CS with the scan rate (**b**), and the specific capacitance (C_sp_) as a function of the scan rate (**c**).

The electrochemical properties of the ceramic supercapacitor electrodes based on the NiMoP coating with rGO and PPy were further studied by the CV method in 0.5 M of Na_2_SO_4_ electrolyte in a potential window from −0.1 to 0.7 V. Figure 6a presents a comparison of the CV curves obtained at 50 mV s^−1^ for the CS substrate modified with PPy as a function of the GO reduction. The NiMoP coated ceramic (CS) substrate exhibits a very low current response, indicating no electrochemical activity. The increase in the current response of the CS electrode upon modification with PPy confirms the synthesis of PPy at the surface of the NiMoP coated ceramic and its potential for energy storage; however, the shape of the CV curve indicates a poor morphology of the PPy coating, allowing the penetration of electrolyte ions to further attack the underlying NiMoP coating. In contrast, the GO-modified PPy coatings exhibit a roughly rectangular shape, which indicates a good potential for achieving an energy storage electrode material [23,50]. The current response and therefore the corresponding capacitance of the electrodes varied in the order PPy/GO < PPy/rGO_E_ < PPy/rGO_C_, which is associated with the improved contribution from rGO_C_ and effect of its improved conductivity [35] on the electrodeposition and surface morphology properties of PPy, as they are known to play a relevant role in the capacitive behavior due to EDLCs and the pseudocapacitive mechanism [51]. It is therefore confirmed that the NiMoP electroless coating can be employed to design ceramic supercapacitor electrodes [23,52].

Figure 6b shows the CV curves of the PPy/rGO_C_ NiMoP coated ceramic electrode with varying scan rates. The electrochemical analysis shows a good current response with almost no deterioration of the CV shape, which can be attributed to the synergetic effect of the electrode material components. The PPy/rGO_C_ electrode exhibits a capacitance of 32.77 mF cm^−2^ at 2 mV s^−1^, which is comparable with other conducting polymer-coated insulating substrates [31]. The performance of the PPy/rGO_C_ electrode may be due to presence of rGO (reduced by the chemical process) that modified the morphology of the PPy coating by creating a complex network and improved its areal capacitive performance by a faster electron transfer and ion diffusion [52].

The area-specific capacitance decreases with increasing the scan rate from 2 to 100 mV s^−1^, as shown in Figure 6c [50,51], which is attributed to the enhanced penetration of the electrolyte ions at lower scan rates, thus allowing them to react with the all the active surface area; meanwhile, at high scan rates, the electrolyte ions is diffusion limited and time constrained to the porous structure of the electrode. The best performance was exhibited by the PPy/rGO_C_ NiMoP coated ceramic with about 660 F g^−1^, the average loading of the electrode material being 0.05 +/− 0.01 mg cm^−2^.

**Figure 7:**
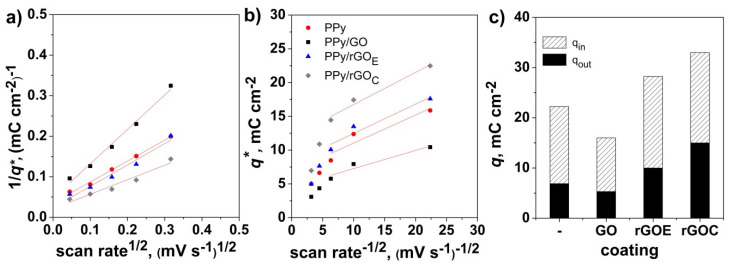
Inverse voltammetric charge vs. the square root of the scan rate (**a**), voltammetric charge vs. the inverse square root of the scan rate (**b**), and the contribution distribution to the total charge from the inner (qin) and outer (qout) charges (**c**) as a function of the electrode material.

From the evolution of the voltammetric charge with the scan rate, one could differentiate the capacitance contribution to the “outer” surface redox and the “inner” redox contributions, according to Trasatti, by considering that the outer surface charge *q_out_* is independent of the scan rate and that the total charge *q* in the bulk of the electrode changes linearly with the square root of the scan rate [17]. The diffusion-controlled charge contribution can be obtained from the difference between the total and the surface charge, which is *q_in_* = *q_t_* − *q_out_*. Figure 7 depicts the Trasatti plots, from where the intercepts were used to find the *q_t_* and *q_out_* and the differentiated capacitance contributions. It is shown that the use of GO affects the electrodeposition and morphology of the PPy coating by forming complex networks and, consequently, both its bulk and surface contribution to the total stored charge. Thus, while the charge contributions decrease when PPy is deposited onto GO, the PPy coating electrodeposited on the rGO exhibits an increased inner charge that can be attributed to the increased conductivity, reduced diffusion path length, and increased accessible surface area due to the stronger interaction between rGO and the polymer matrix, similarly to other reports [53,54,55], as well as increased surface charge, which can be attributed to the increased active sites at the surface.

**Figure 8:**
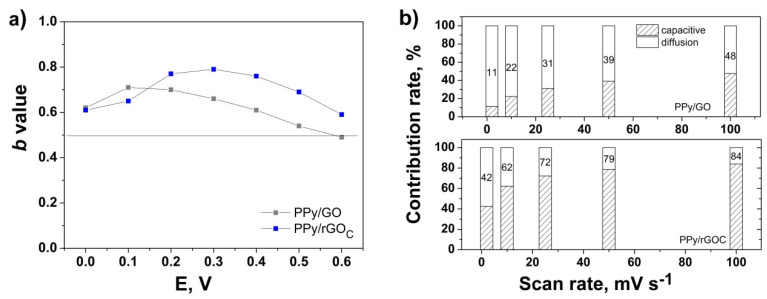
Evolution of the b value with the potential (**a**) and contributions to the current response with the scan rate (**b**) for PPy electrodeposited onto GO and rGO_C_.

The electrode kinetics may be studied with applied potential according to Conway et al. by using the equation *i(V) = aυ^b^,* where *i* represents the current at a given potential V, *υ* represents the scan rate, and *a* and *b* are adjustable parameters [18]. By evaluating the value of *b* from the slope of the linear fit of log *i* vs. log *υ* plot at a given potential, one can differentiate the diffusion-limited systems and surface-controlled storage ones [18,56,57]. The intercalation of ions into the electrode or diffusion-controlled process is indicated by b = 0.5, while b = 1 represents a capacitive process via a surface faradaic redox reactionor non-diffusive controlled process [19,58]. Figure 8a presents the effect of GO on the *b* value for the oxidation CV curve of the electrodeposited PPy. The calculated *b* values are above 0.5 in the investigated potential range for the modified PPy coating with respect to the unmodified PPy, which showed a b value of below 0.5 (not shown). The modified PPy showed a higher *b* value upon the reduction of GO of up to 0.8, which indicates a higher dominance on the charge storage mechanism from the surface capacitive process in comparison to the diffusion-limited process, which can be attributed to the improved electrodeposition of PPy onto rGO. The difference, with respect to the Trasatti evaluation, is attributed to the varying assumptions of each method. However, to better understand the surface capacitive contribution, the fraction of current response was analyzed as the scan rate increased from 2 to 100 mV s^−1^, according to Dunn, by using the equation *i(V) = k_*1*_υ + k*2*υ^*1/2*^* derived to *i(V)/υ^*1/2*^ = k_*1*_ υ^*1/2*^ + k*2**, where the terms *k_*1*_υ + k*2*υ^*1/2*^* are attributed to the surface capacitive process and the diffusion-controlled contribution, respectively [19,59]. The values of *k*_1_ and *k*_2_ obtained from the slopes and intercepts of the plot of the derived equation are further used to calculate the contributions of the two elements. Figure 8b depicts the corresponding contributions to the response at a given potential of 0.3 V at various scan rates. It is shown that the proportion of surface capacitive process upgrades with the scan rate, reaching 84% at 100 mV s^−1^ for the PPy electrodeposited on rGO_C_. The 2 to 3-fold higher capacitive contribution of PPy upon the reduction of GO may accounted for the enhanced conductivity of the coating and more redox-active sites [60]. The diffusion-limited contribution reduced gradually with the increasing scan rate as the ion insertion gets impeded at higher scan rates, consistent with previous reports [61,62,63].

**Figure 9:**
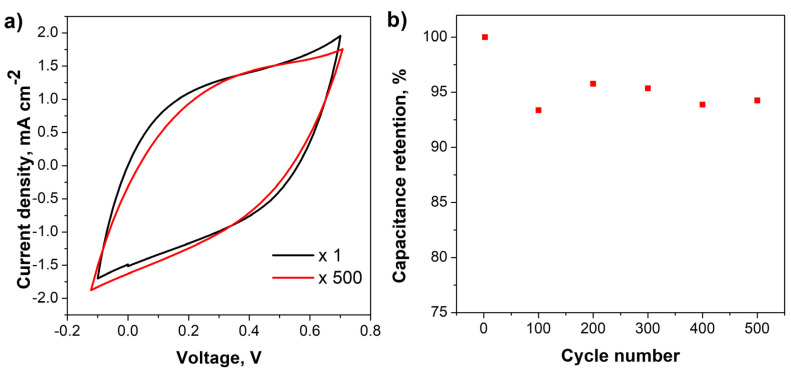
CV curves of PPy/rGO_C_–CS at 50 mV s^−1^ at 1 and 500 cycles (**a**) and areal capacitance as a function of the cycle number (**b**).

For practical applications in supercapacitors, cycling stability analysis is an important and critical parameter to take into consideration [34,64]. Thus, the electrochemical cycling stability of the PPy/rGO_C_ electrode was evaluated at 50 mV s^−1^ for 500 cycles, as shown in Figure 9. The CV shape and electrochemical current response of the PPy/rGO_C_ electrode is almost similar after 500 cycles, as indicated in Figure 9a. The retention of areal-specific capacitance depicted in Figure 9b shows an excellent retention value of 94% upon 500 charge/discharge cycles, which could be attributed to the synergetic effect of GO reduced by ascorbic acid and PPy [65]. Such results indicate the excellent adhesion and stability of the coating upon cycling.

## 4. Conclusions

The surface properties of the ceramic substrate were modified by a simple combined approach based on electroless deposition and electrodeposition. The deposition and properties of the PPy film were analyzed with the use of a GO film as a primer before electrodeposition. The results indicate that the electroless NiMoP film allows the successful electrodeposition of PPy-based coatings. The reduction degree in the GO film obtained by the chemical reduction with ascorbic acid markedly improved the deposition in terms of morphology and, consequently, its surface capacitive contribution towards the total stored charge. The electrode obtained by the electrodeposition of PPy onto chemically reduced GO Ni-Mo-P-supported ceramic reached about 660 F g^−1^ at 2 mV s^−1^ and exhibited a 94% capacitance retention upon 500 charge/discharge cycles at 50 mV s^−1^. This work demonstrates that the proposed approach is viable for conferring ceramic substrate with functionality and offers an insight into the effect of GO addition on the capacitance contribution to the total stored charge, which should be useful for the design of ceramics for next-generation energy storage devices.
